# Enhancing gait training with anti-gravity treadmill ‘Alter-G’ in patients with Parkinson’s disease

**DOI:** 10.1371/journal.pone.0341021

**Published:** 2026-02-25

**Authors:** Desireè Latella, Antonino Lombardo Facciale, Mirjam Bonanno, Lilla Bonanno, Antonino Leo, Roberta Cellini, Giuseppe Di Lorenzo, Piero Buonasera, Caterina Formica, Giuseppe Paladina, Luca Pergolizzi, Bartolo Fonti, Angelo Quartarone, Rocco Salvatore Calabrò

**Affiliations:** I.R.C.C.S. Centro Neurolesi Bonino Pulejo, Messina, Italy; University of Catania, ITALY

## Abstract

**Background:**

Freezing of Gait (FoG) is a debilitating motor symptom affecting nearly half of individuals with Parkinson’s disease (PD), increasing fall risk and reducing independence. Despite pharmacological and neuromodulation therapies, residual symptoms often persist, underscoring the need for complementary rehabilitation strategies.

**Objective:**

To evaluate the effects of body weight–supported treadmill training using differential air pressure on gait and mobility in PD patients.

**Methods:**

Forty patients with idiopathic PD were randomly assigned to an experimental group (EG, n = 20), who received gait training with a lower-body positive pressure treadmill, or to a control group (CG, n = 20), who underwent conventional physiotherapy. Both groups trained twice weekly for 4 months. Outcomes were assessed at baseline (T0) and post-intervention (T1), and classified as primary (gait speed, step length, balance, postural stability) or secondary (quality of life, fear of falling, anxiety).

**Results:**

Compared to controls, the EG showed significant improvements in gait speed, step length, balance, and postural stability (all p < 0.01). Secondary outcomes also improved, with reductions in fear of falling (p < 0.01) and better quality of life scores (p < 0.01).

**Conclusions:**

This pilot trial suggests that body weight–supported treadmill training through differential air pressure may improve gait performance and postural control, while also enhancing quality of life in PD patients with mobility impairments and FoG. These preliminary findings support its potential role as a complementary rehabilitation strategy, warranting confirmation in larger trials.

## 1. Introduction

Parkinson’s disease (PD) is the second most common neurodegenerative disorder, with an estimated prevalence and incidence in Europe of approximately 108–257 per 100,000 and 11–19 per 100,000 persons per year, respectively [1]. Clinically, PD is characterized by a combination of motor symptoms, including bradykinesia, tremor, rigidity, and postural instability, and a wide range of non-motor symptoms, such as apathy, dysphoria, cognitive impairment, sleep disorders, autonomic dysfunction, and mood disturbances [2,3]. Among the motor manifestations, gait disturbances and balance impairments represent some of the most disabling features of PD, contributing significantly to falls, loss of independence, and reduced quality of life. One of the most severe and complex gait disturbances is freezing of gait (FoG), an episodic phenomenon defined as a brief, involuntary inability to initiate or continue walking, typically occurring during gait initiation, turning, or navigating through narrow spaces [4,5]. FoG affects nearly 47% of PD patients [2] and is strongly associated with an increased risk of falls and reduced functional mobility. In rare cases, patients may remain immobile for several minutes until external cues help them resume movement [4,6]. Despite the availability of pharmacological therapies and neurostimulation techniques, FoG remains particularly challenging to manage. Many patients do not respond adequately to dopaminergic medications or require invasive and costly interventions [7,8]. As a result, there is growing interest in non-pharmacological treatments, especially physiotherapy-based approaches. Treadmill training, cueing strategies, and aquatic therapy have shown promise due to their non-invasive nature and potential benefits on gait parameters, cardiovascular health, and quality of life [7]. Additionally, body weight support (BWS) systems have been widely used in the context of motor neurorehabilitation. BWS during treadmill training can reduce lower-limb load, allowing early, intensive gait practice with lower energy cost and less joint stress, while preserving cardiorespiratory fitness [9,10]. By minimizing joint stress and fall risk, it may also be particularly beneficial for patients with musculoskeletal pain syndromes [11,12]. Rhythmic, cyclic stepping under BWS can activate spinal central pattern generators, facilitate hip extension and faster gait, and promote compensatory activation of supplementary motor and premotor areas that support neuroplastic reorganization [13]. In this context, the Anti-Gravity Treadmill, such as Alter-G, emerges as an innovative rehabilitation device that may enhance gait training in PD. Using differential air pressure technology to reduce body weight during ambulation, anti-gravity treadmill allows for safer and more controlled gait training, facilitating step initiation and improving walking dynamics.

This randomized controlled trial aims to evaluate the effectiveness of BWS treadmill training on gait performance in individuals with PD, with a primary focus on FoG episodes and gait cycle quality. Secondary outcomes include cadence, step length, walking speed, pain, mood, and overall quality of life, providing a comprehensive assessment of the intervention’s therapeutic potential.

## 2. Materials and methods

### 2.1 Study design and population

**Fig 1:**
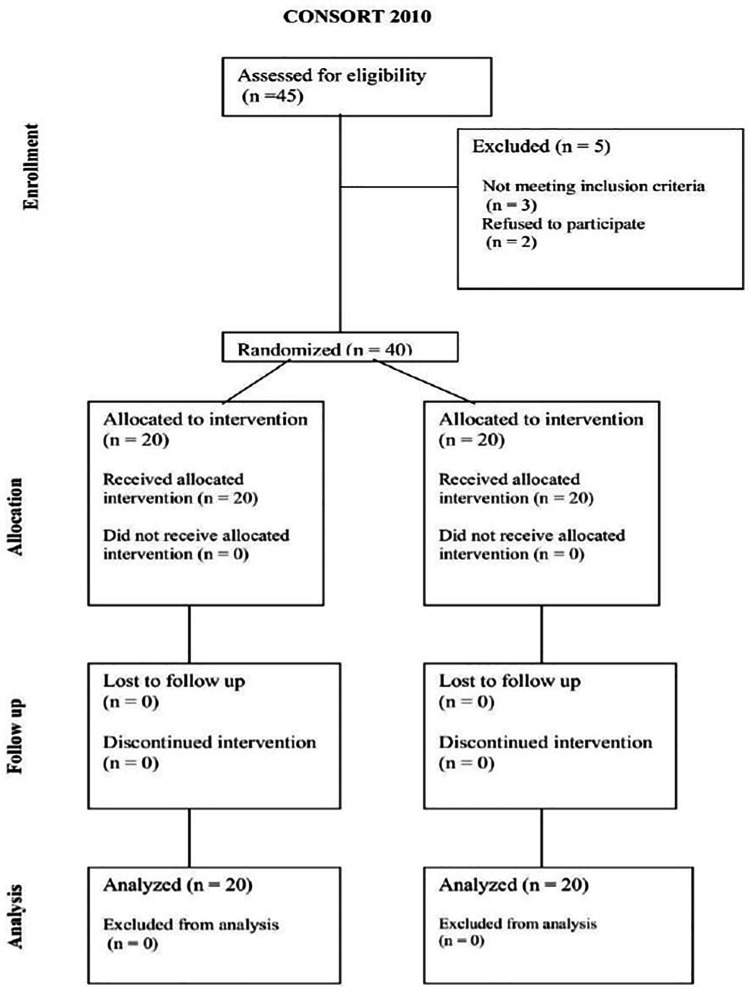
CONSORT 2010 flow diagram of the study. Of the 45 patients assessed for eligibility, 5 were excluded (3 did not meet inclusion criteria, 2 declined to participate). Forty patients were randomized in a 1:1 ratio to the experimental group (n = 20) or the control group (n = 20). All patients received the allocated intervention, no losses to follow-up or discontinuations occurred, and all participants were included in the final analysis.

A priori sample size calculation was not performed, as this study was designed as an exploratory pilot randomized controlled trial. The primary aim was to generate preliminary data to inform future large-scale studies and formal power calculations. A total of 45 patients diagnosed with PD who had a Unified Parkinson’s Disease Rating Scale (UPDRS) score of ≤81. Patients were enrolled from attended Parkinson’s Outpatient Clinic at the IRCCS Centro Neurolesi “Bonino-Pulejo” (Messina, Italy) from May 2021 to March 2023 (Clinical Trial: NCT0574025) (https://clinicaltrials.gov/). Of these, 5 were excluded (3 did not meet the inclusion criteria and 2 declined to participate), leaving 40 patients who were enrolled in the trial. Eligibility was based on predefined inclusion and exclusion criteria. (Fig 1)

#### Inclusion criteria.

Diagnosis of PD according to the “Movement Disorder Society Clinical Diagnostic Criteria for Parkinson’s Disease”; evidence of FoG; age between 50 and 85 years; Hoehn and Yahr stage between II and III; Mini Mental State Examination (MMSE) score ≥ 24 (age- and education-corrected); no musculoskeletal disorders; no visual or auditory impairments; no known cardiovascular or pulmonary diseases.

#### Exclusion criteria.

Diagnosis of parkinsonism according to the “Movement Disorder Society Clinical Diagnostic Criteria for PD”; presence of a pacemaker or infusion systems (e.g., Duodopa gel via J-Peg system); comorbidities (cardiac, pulmonary, or musculoskeletal) contraindicating physical exercise; severe visual or auditory impairment.

After baseline assessment, participants were randomly assigned in a 1:1 ratio to the Experimental Group (EG; n = 20), who received body weight–supported treadmill training using differential air pressure in combination with conventional therapy, or to the Control Group (CG; n = 20), who underwent only traditional physical exercise. Randomization was performed using a computer-generated simple random sequence (random.org). No stratification or block randomization procedures were applied. Because of the nature of the intervention, blinding of patients and therapists was not feasible; however, all outcome assessments were performed by evaluators blinded to group allocation in order to minimize detection bias.

The Local Ethics Committee of IRCCS Centro Neurolesi “Bonino Pulejo” approved the study (U224/22), participants gave their informed consent signed.

### 2.2 Procedures and device

Each participant underwent neuropsychological and motor evaluation at baseline (T0) and after the treatment (T1). The 20 patients of CG performed a traditional gait training program using a conventional treadmill, adhering to a standard approach for improving walking. The 20 patients of EG performed a gait training program with Alter-g anti-gravity treadmill. All patients were supervised by two physiotherapists on-site, who, after an initial thorough physiotherapeutic assessment, developed a personalized rehabilitative program, for a total of 20 sessions (2 times a week for 4 months). Each session, lasting approximately 60 minutes, comprised three phases: warm up, treadmill training (traditional or anti-gravity), and cool down. The warm-up phase, lasting approximately 15 minutes, aims to prepare the body for treadmill training by enhancing performance and reducing injury risk, increasing body temperature, heart rate, joint lubrication, and blood flow, while reducing stress and fatigue. The active training phase utilized an Alter-G anti-gravity treadmill for the experimental group and a traditional treadmill for the control group, with both groups walking barefoot on a treadmill set to zero-degree incline. Participants were instructed to maintain proper posture, adjust their gait to the treadmill speed, and safely end the session if necessary. Initial treadmill speeds were based on baseline gait analysis and were gradually increased over 20 sessions to reach each participant’s maximum walking capacity while ensuring proper gait performance. In the experimental group, a 60% body weight reduction was applied during each session using the Alter-G system. After treadmill training, all participants performed a 15-minute cool-down phase, including low-intensity walking and muscle stretching, to support both physiological and mental recovery. The EG underwent gait training using the AlterG anti-gravity treadmill model M320 (AlterG, Inc., Fremont, CA, USA).

### 2.3 AlterG system

[14] The body weight–supported treadmill system used in this study relies on lower-body positive pressure (LBPP) technology, which provides unloading of the body through differential air pressure. Once the patient is enclosed in a waist-height chamber, the system calibrates body weight and applies positive air pressure to reduce the effective load on the lower limbs. This allows precise adjustment of support from 20% to 100% of body weight, typically in 1% increments. The mechanism enables patients to walk safely while maintaining near-physiological gait kinematics, reducing joint stress, and lowering the risk of falls. Training intensity can be progressively increased by modulating treadmill speed and the percentage of body weight supported, thereby ensuring individualized and safe rehabilitation.

In this trial, the experimental group performed training using the Alter-G Anti-Gravity Treadmill (model M320, AlterG Inc., Fremont, CA, USA). [14].

### 2.4 Outcome measures

Primary and secondary outcomes were defined a priori. Primary outcomes focused on motor performance and balance, while secondary outcomes assessed quality of life, fear of falling, and anxiety.

#### 2.4.1 Primary outcomes.

The primary endpoints of the study were functional motor outcomes related to balance, mobility, and gait performance. These were assessed using:

- Berg Balance Scale (BBS) consists of 14 mobility tasks, with tasks varying in levels of difficulty. Tasks are divided into 3 domains, namely seated balance, standing balance, and dynamic balance. Each activity is assessed on a Likert scale of 5 points with a maximum score of 56. The total score determines the predicted fall risk. Overall, total scores below 45 are associated with a higher risk of falls. An individual with a history of falls and a total score below 51 is highly predictive of falls. A score below 40 is associated with almost a 100% risk of falling [15].
- 6-minute Walk Test (6MWT) assesses the distance covered in 6 minutes and provides information about endurance, and cardiorespiratory function and evaluate the response to therapeutic treatments [16]Timed Up and Go (TUG) provides an observational approach to gait assessment and can help to predict risk of falls. The test assess time to rise from a chair, walk 3 meters, turn around, and then sit down again. It has been suggested that a cut-off point of 13.5 seconds may identify individuals an increased risk of falls [17].
- 10-Meter Walk Test is a performance measure used to assess gait speed in meters per second over a short distance. The total time taken to walk 6 meters is recorded [18].
- The Tinetti Gait and Balance Test assesses a patient’s balance and gait using a standardized scoring system. The scoring system is ordinal, with a range from zero to two. A score of zero indicates severe impairment, while a score of two signifies independence. If a patient scores 18 or lower, they are at high risk of falling. Conversely, patients scoring between 19 and 23 have a moderate risk of falling, and those scoring 24 or higher have a statistically low risk of falling [19].
- Postural stability was evaluated using the Zebris Medical GmbH platform, a pressure-sensitive system that records parameters such as Center of Pressure (CoP) length, average velocity, 95% confidence ellipse, and load distribution. Patients were assessed standing still with eyes open and closed for 10 seconds. These measures provide insights into balance, postural control, and load symmetry. Higher CoP length or velocity indicates instability, while a smaller confidence ellipse suggests better stability. Load symmetry close to 50% reflects balanced posture, while deviations indicate potential imbalances [21].
- Gait parameters were analyzed at T0 and T1 using the BTS Gaitlab, equipped with motion capture systems, force platforms, and electromyographic sensors. Following the DAVIS protocol, reflective markers and EMG sensors were placed on key anatomical points. Patients performed six walking trials at a comfortable speed, and parameters such as cadence, step width, step length, stance and swing phases, average speed, Gait Deviation Index (GDI), and Gait Profile Score (GPS) were recorded. GDI and GPS quantify gait quality, with higher GDI and lower GPS indicating patterns closer to normal. The stance phase (60% of the gait cycle) and swing phase (40%) were analyzed for stability and efficiency, while cadence, step width, and step length offered further insights into walking performance. The data, collected through motion capture and stabilometric systems, allowed a comprehensive assessment of postural stability and gait deviations, supporting the evaluation of therapeutic interventions and tracking progress over time [22].

#### 2.4.2. Secondary outcomes.

Secondary endpoints were related to psychological and quality-of-life dimensions, as well as perceived risk of falls:

- The Falls Efficacy Scale International (FES-I) is a measure of “fear of falling” or “concerns about falling,” based on the operational definition of this fear as “low perceived self-efficacy in avoiding falls during essential and non-dangerous daily activities.” It is a 16-item questionnaire with scores ranging from a minimum of 16 (no concern about falling) to a maximum of 64 (strong concern about falling). The FES-I appears to be a reliable and valid method for assessing fear of falling [20].
- The Parkinson’s Disease Questionnaire-39 (PDQ-39) is a widely used, disease-specific instrument designed to assess health-related quality of life (HRQoL) in individuals with PD. The questionnaire consists of 39 items grouped into eight domains, which evaluate different aspects of daily living and well-being affected by the disease. Each item is scored on a 5-point Likert scale ranging from 0 (never) to 4 (always), reflecting the frequency or severity of issues experienced in the past month. The scores for each domain are summed and transformed into a scale from 0 to 100, where higher scores indicate a greater negative impact on quality of life [23].
- The EuroQol-5 Dimension (EQ-5D) is a standardized instrument used to measure health-related quality of life (HRQoL) across various populations and conditions, including individuals with PD. Developed by the EuroQol Group, it provides a simple and generic measure of health status that is applicable in clinical and research settings. In the context of PD, it complements disease-specific tools like the PDQ-39 by offering a generic perspective on overall health and well-being [24].
- The Hamilton Anxiety Rating Scale (HAM-A) is one of the most widely used clinician-administered tools for assessing the severity of anxiety symptoms. It consists of 14 items, each addressing a specific symptom or cluster of symptoms commonly associated with anxiety. Each item is scored on a 5-point scale ranging from 0 (not present) to 4 (very severe). The total score ranges from 0 to 56. Higher scores indicate greater severity of anxiety symptoms [25].

### 2.5 Gait analysis

Participants in the experimental group underwent quantitative gait analysis at baseline (T0) and after treatment (T1) using the BTS Gait Lab (BTS Bioengineering, Milan, Italy). The system includes infrared cameras, force plates, and wireless electromyographic sensors. Data acquisition followed the DAVIS multifactorial protocol, which combines kinematic, kinetic, and electromyographic assessments.

Reflective markers were placed on standard anatomical landmarks, and surface EMG electrodes were applied to major lower-limb muscles. Each participant performed standing and walking trials at a self-selected comfortable speed.

The main outcomes extracted were Kinematics: hip, knee, ankle, and pelvic joint angles; Kinetics: joint moments and powers; Electromyography: timing of activation/deactivation of tibialis anterior, gastrocnemius, quadriceps, and hamstrings; Spatiotemporal parameters: cadence, step width, step length, stance and swing phases, gait speed; Indices of gait quality: Gait Deviation Index (GDI) and Gait Profile Score (GPS).

This protocol provided an integrated evaluation of motor performance and changes in gait quality following the intervention.

## 3. Statistical analysis

Descriptive analysis was reported for demographic and clinical variables. Continuous variables were expressed as mean ± standard deviation, whereas categorical variables in frequencies and percentages. For continuous data, the assumption of normality was assessed using the Shapiro-Wilk test. Normally distributed data were analyzed using parametric tests, while non-normally distributed data were analyzed using non-parametric tests applied directly to the raw data. Specifically, u npaired Student’s t-test or Mann-Whitney U test was used for inter-group analysis, while paired Student’s t-test or Wilcoxon signed rank test for intra-group analysis. Between-group comparisons were performed at both T0 and T1, while within-group comparisons assessed changes over time. We did not analyze raw change scores (Δ = T1-T0), as these are known to inflate error variance in small samples and were not aligned with the primary objective of our study. We considered Δ to calculate Spearman’s rank correlation to explore potential associations between QoL, gait analysis scores and motor scale. Analyses were performed using an open source R4.2.2 software package. A 95% of confidence level was set with a 5% alpha error. Statistical significance was set at p < 0.05. Given the small sample size and multiple endpoints, p-values must be interpreted with caution.

## 4. Results

### 4.1 Inter-group analysis

Baseline demographic and clinical characteristics of the two groups are reported Tables 1 and 2. No significant differences were found in age (p = 0.25), sex distribution (p = 0.99) and education level (p = 0.99) between the EG and CG, indicating overall homogeneity at baseline. However, significant baseline differences were observed in some PDQ-39 subdomains in Mobility (p = 0.01), cognitive and communicative abilities were also significantly different, with Cognition (p = 0.01) and lower Communication (p = 0.03) scores in the experimental group. Additionally, motor performance assessments such as the 10-Min Walk Test (p < 0.001), as well as gait and balance parameters (Stance Phase Righ/Left, Swing Phase Right/Left and Gait Speed all p < 0.001), confirmed the initial functional disparities between the two groups. The experimental group also exhibited significantly lower Gait Deviation Index Left (p = 0.02), reflecting a difference in movement patterns compared to controls. At T1, new significant differences emerged, with the experimental group showing greater improvement in EQ5-D (p = 0.005) and Mobility (p = 0.007) suggesting a positive impact on quality of life and mobility in the experimental group. Improvements were also observed in Communication (p < 0.001). Motor performance further differentiated the two groups at T1, with significant differences in FES-I (p < 0.001), 10-MWT (p < 0.001), TUG Right, TUG Left, and BBS (p < 0.001), confirming better functional mobility and balance in the experimental group. UPDRS-III (p < 0.001), HY (p < 0.001) and the 6-MWT (p < 0.001) reflected further motor improvements. Finally, several gait and postural parameters, including Cadence, Average Gait Speed, Stride width, Gait Deviation Index Right/Left, Gait Profile Score Right/Left, Step Length Right/Left (p < 0.001) showed significant improvements in the experimental group compared to controls.

**Table 1:** Demographic characteristics of the study sample.

	Global sample	EG	CG
N. subjects	40	20	20
Male	30 (75%)	15 (75%)	15 (75%)
Female	10 (25%)	5 (25%)	5 (25%)
Age years (Mean±SD)	67.18 ± 7.29	68.5 ± 7.75	65.9 ± 6.73
Education (Mean±SD)	2.73 ± 0.99	2.70 ± 1.03	2.75 ± 0.97
Disease Duration (Mean±SD)	12 ± 8	9 ± 8.5	12.5 ± 7.5

**Table 2:** Primary motor outcomes measure at T0 (baseline) and T1 (post-treatment). Data are presented as means ± standard deviations for normally distributed variables and medians (first and third quartiles) for non-normally distributed variables.

		Experimental	Control	p-value
10MWT	T0	5.7 (5.3-7.8)	5.6 (5.0-7.4)	<0.001^µ*^
	T1	5.8 (4.2-6.4)	5.6 (5.0-7.4)	<0.001^µ*^
	p	0.01^¥*^	–	
TUG- Right	T0	12.3 (10.1-14.7)	11.3 (8.9-13.4)	0.26^µ^
	T1	10.9 (8.7-14.2)	11.3 (8.9-13.4)	<0.001^µ*^
	p	0.003^¥*^	–	
TUG- Left	T0	12.9 (9.6-15.2)	11.1 (9.6-13.2)	0.25^µ^
	T1	11.3 (8.9-13.5)	11.1 (9.6-13.2)	<0.001^µ*^
	p	0.003^¥*^	–	
BBS	T0	47.5 (36.5-50.2)	44.0 (40.0-47.2)	0.43^µ^
	T1	50.0 (44.0-54.0)	44.0 (40.0-47.2)	<0.001^µ*^
	p	0.001^¥*^	–	
6MWT	T0	300. ± 75.6	280.0 (217.5-306.7)	0.15^µ^
	T1	343.7 ± 87.3	280.0 (217.5-306.7)	<0.001^µ*^
	p	0.0003^± *^	–	
TS- B	T0	14.0 (12.5-15.0)	12.0 (10.0-14.0)	0.06^µ^
	T1	15.0 (13.7-16.0)	12.0 (10.0-14.0)	<0.001^µ*^
	p	0.03^¥*^	–	
TS- G	T0	9.0 (7.0-10.2)	8.0 (6.7-10.0)	0.28^µ^
	T1	10.0 (8.7-11.0)	8.0 (6.7-10.0)	<0.001^µ*^
	p	0.02^¥ *^	–	

^±^paired Student’s t-test (parametric test).

^¥^Wilcoxon signed rank test (nonparametric test).

^µ^Mann-Whitney U test (nonparametric test).

Legend: 6MWT = 6-Minutes Walk Test; 10MWT = 10-Meters Walk Test; BBS = Berg Balance Scale; TS-B = Tinetti Scale – Balance; TS-G = Tinetti Scale – Gait; TUG = Timed Up and Go Test.

### 4.2 Intra-group analysis

Within the experimental group, a detailed intra-group analysis revealed significant improvements in multiple aspects from baseline (T0) to post-intervention (T1). Quality of life and overall functional independence improved significantly, as reflected in the increase in EQ5-D (p = 0.03), suggesting that participants perceived a better health status after the intervention. Motor performance showed clear advancements. The decrease in 10-MWT time (p = 0.01) and in TUG Right (p = 0.003) and TUG Left (p = 0.003) indicated faster walking speed and improved mobility. Additionally, a significant increase in BBS (p = 0.001) confirmed enhanced balance and postural control. Furthermore, an increase in the 6-MWT (p = 0.0003) highlighted better endurance and walking capacity. Gait and locomotion parameters followed the same positive trend. TS-B (p = 0.03) and TS-G (p = 0.02) increased, reflecting better step control and gait efficiency. Walking speed, measured by Average Gait speed also increased (p = 0.02), along with improvements in Stride length, as shown by increases in Step Length Right (p = 0.001) and Step Length Left (p = 0.004). Postural stability showed positive changes as well. A decrease in Ellipse (p = 0.04) and Path length CoP (p = 0.04) suggested improved balance, with reduced body sway and better control over posture. In contrast, the control group did not show any significant improvements between T0 and T1, indicating that the observed changes in the experimental group were likely due to the intervention.

### 4.3 Correlation analysis

**Fig 2:**
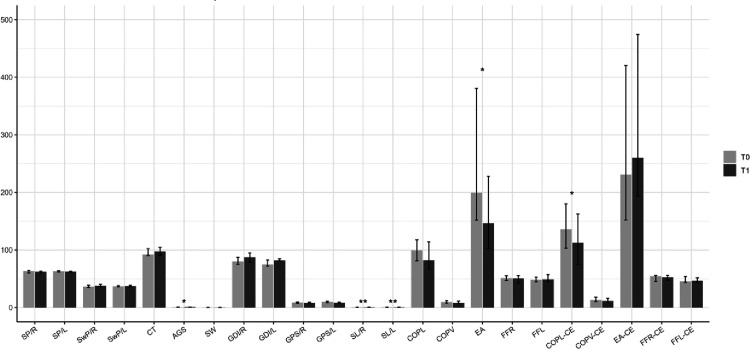
Comparison between baseline (T0) and follow-up (T1) values in the experimental group for gait and balance variables. Bars represent the median, error bars indicate the interquartile range (IQR). Asterisks denote statistically significant differences between T0 and T1 (*p < 0.05, **p < 0.01).Legend: SP/R = Stance Phase Right; SP/L = Stance Phase Left; SwP/R = Swing Phase Right; SwP/L = Swing Phase Left; CT = Cadence; AGS = Average Gait Speed; SW = Step Width; GDI/R = Gait Deviation Index Right; GDI/L = Gait Deviation Index Left; GPS/R = Gait Profile Score Right; GPS/L = Gait Profile Score Left; SL/R = Step Length Right; SL/L = Step Length Left; CoPL = Center of pressure- Path length; CoPV = Center of pressure- Average velocity; EA = Ellipse; FFR = Foot Force Right; FFL = Foot Force Left; CoPL-CE = Center of pressure- Path Length- closed eyes; CoPV- CE = Center of pressure- average velocity- closed eyes; EA- CE = Ellipse- closed eyes; FFR- CE = Foot Force Right- closed eyes; FFL-CE = Foot Force Left- closed eyes.

In addition to evaluating treatment-related changes, correlation analysis were conducted to explore relationship between patient-reported outcomes and instrumented gait measures within the experimental group. Mobility showed positive associations with Gait Profile Score Right (r = 0.61, p < 0.001) and COP path length (r = 0.49, p = 0.03), while Emotional Well-Being correlated with step length left (r = 0.62, p < 0.001) and right showed a similar trend (r = 0.45, p = 0.05). Swing-phase metrics were consistently related to spatiotemporal performance (Swing Phase Left with cadence and average gait speed; all r ≥ 0.53, p ≤ 0.02). In contrast, Mobility correlated inversely with gait deviation right (r=−0.64, p < 0.001), ADL was negatively related to Swing Phase Right (r=−0.59, p = 0.01) and cadence was inversely associated with COP path length (r=−0.52, p = 0.02) Fig 2. Full results are provided in Supplementary S1 Fig, correlation matrix with significance Table 3.

**Table 3:** Secondary outcomes measure at T0 (baseline) and T1 (post-treatment). Data are presented as means ± standard deviations for normally distributed variables and medians (first and third quartiles) for non-normally distributed variables.

		Experimental	Control	p-value
FES-I	T0	26.5 (17.7-37.2)	25.0 (23.0-41.5)	0.51
	T1	22.0 (17.0-36.2)	25.0 (23.0-41.5)	<0.001^µ*^
	p	0.09^¥^	^–^	
EQ-5D	T0	50.0 (50.0-60.0)	50.0 (50.0-60.0)	0.60^µ^
	T1	60.0 (50.0-60.0)	50.0 (40.0-50.0)	0.005^µ *^
	p	0.03^¥ *^	0.12^¥^	
PDQ-39 Mobility	T0	32.5 (18.1-55.0)	62.0 ± 20.14	0.01^* µ^
	T1	27.5 (15.6-47.5)	62.0 ± 20.14	0.007^µ *^
	p	0.42^¥^	–	
PDQ-39 ADL	T0	24.9 (16.6-49.9)	33.3 (29.2-59.4)	0.09^µ^
	T1	22.9 (14.5-56.2)	33.3 (29.2-59.4)	0.07^µ^
	p	0.67^¥^	–	
PDQ-39 Emotional Well-being	T0	31.2 (25.0-45.8)	50.0 (25.0-70.8)	0.07^µ^
	T1	39.5 (16.7-55.2)	50.0 (25.0-70.8)	0.11^µ^
	p	0.61^¥^	^–^	
PDQ-39 Stigma	T0	25.0 (12.5-32.8)	25.0 (6.2-37.5)	0.79^µ^
	T1	25.0 (12.5-32.8)	25.0 (6.2-37.5)	0.79^µ^
	p	0.34^¥^	^–^	
PDQ-39 Social Support	T0	0 (0-8.3)	8.3 (0-35.4)	0.05^µ^
	T1	0 (0-10.4)	8.3 (0-35.4)	0.05^µ^
	p	0.27^¥^	–	
PDQ-39 Cognition	T0	43.7 (35.9-59.4)	35.3 ± 10.9	0.01^µ *^
	T1	50.0 (29.7-7.31)	35.3 ± 10.9	0.06^µ^
	p	0.50^¥^	–	
PDQ-39 Communication	T0	20.8 (0-33.3)	36.7 ± 24.1	0.03^µ *^
	T1	25.0 (6.2-41.7)	36.7 ± 24.1	0.0007^µ *^
	p	0.37^¥^	–	
PDQ-39 Bodily Discomfort	T0	19.1 ± 25.8	50.0 (41.6-52.1)	0.57^µ^
	T1	50.0 ± 23.6	50.0 (41.6-52.1)	0.60^µ^
	p	0.79^±^	–	
HAM-A	T0	15.7 ± 8.6	18.0 (10.5-21.0)	0.77^µ^
	T1	14.3 ± 7.9	17.0 (11.0-21.0)	0.32^µ^
	p	0.12^±^	0.26	

^±^paired Student’s t-test (parametric test).

^¥^Wilcoxon signed rank test (nonparametric test).

^µ^Mann-Whitney U test (nonparametric test).

Legend: FES-I = Falls Efficacy Scale International; EQ-5D = EuroQol-5 Dimensions; HAM-A = Hamilton Anxiety Rating Scale PDQ-39 = Parkinson’s Disease Questionnaire – 39 items.

## 5. Discussion

Rehabilitation in PD aims to improve motor symptoms, psychological well-being, and overall quality of life. In this context, gait training with body weight support through differential air pressure may help maintain motor function, slow disability progression, and enhance emotional and cognitive health [26–28]. From our results, the intervention promoted significant improvements in gait speed, step length, balance, and postural stability, confirming the relevance of targeted motor rehabilitation in this population. Several rehabilitation treatments are commonly employed in PD [29,30]. For instance, conventional physiotherapy, through physical exercises, focuses on flexibility, strength, and balance training to mitigate bradykinesia and rigidity. Moreover, cueing-based therapy, including auditory, visual, and proprioceptive cues, enhances rhythmic movement patterns and compensatory strategies. Innovative technologies such as virtual reality (VR), offer multisensorial feedback and tailored exercises, exploiting visual and auditory cues, with the aim to restore patients’ functional independence [31–33]. Among BWS approaches devices such as Rysen system provide safe gait training sustained trunk stability [13], whereas aquatic therapy provides a low-impact environment for mobility training, reducing fall risk and improving cardiovascular endurance [34]. Compared to these modalities, the LLBP therapy treadmill allows precise, incremental unloading of body weight while preserving natural gait kinematics. By minimizing gravitational load, it facilitates safer and more controlled gait training, promoting motor learning and potentially reducing the severity and frequency of FoG episodes [14]. These mechanisms may support the reorganization of locomotor patterns at the spinal level, and influence central pattern generators thereby restoring a more physiological walking rhythm. Our study demonstrated that body weight–supported treadmill training was associated with significant improvements in postural control, as indicated by reduced ellipse area with eyes open and reduced CoP path length with eyes closed. These findings suggest enhanced balance and proprioceptive integration, corroborated by clinical improvements in BBS and Tinetti scores [35,36]. Improvements in gait speed and step length, validated through gait analysis and functional tests (10MWT, TUG), highlight the positive impact on bradykinesia-related gait disturbances [37,38]. Given that reduced step length is a hallmark of PD-related gait dysfunction, its recovery represents a clinically meaningful outcome associated with reduced fall risk. FES-I scores further confirmed a decrease in patients’ fear of falling, supporting the alignment between objective and subjective measures of fall risk. Beyond motor outcomes, improvements were also observed in health-related quality of life. Gains in PDQ-39 and EQ-5D scores suggest that enhanced mobility and stability may have contributed to better perceived health status and independence. This is particularly relevant as fear of falling and loss of autonomy represent major contributors to psychological distress and reduced participation in PD [39]. The observed correlation between mobility improvements and reduced anxiety reinforces the interdependence between motor and emotional health in this population. These results should be interpreted cautiously. The relatively small sample size (n = 20 per group) limits generalizability, and the exploratory nature of the trial increases the risk of Type I error. In addition, multiple endpoints were assessed, and although appropriate statistical tests were applied, a repeated-measures analysis was not feasible. Larger randomized controlled trials are needed to confirm these preliminary findings and establish long-term effects.

In summary, our findings provide preliminary evidence that body weight–supported treadmill training through differential air pressure may improve gait performance, balance, and perceived fall risk in PD [40]. By addressing both motor and psychosocial dimensions, this approach may represent a valuable rehabilitation strategy, complementing existing therapies such as conventional physiotherapy and aquatic training. Future studies should further investigate its comparative benefits and neurophysiological mechanisms in larger cohorts.

## 6. Conclusion

This exploratory pilot trial showed that body weight–supported treadmill training through differential air pressure improved key primary outcomes (gait speed, step length, balance, and postural stability) and led to improvements in secondary outcomes (quality of life and fear of falling) in patients with PD. These findings support the potential role of this approach as a complementary rehabilitation strategy, to be further validated in larger studies with long-term follow-up.

## Supporting information

S1 Fig Spearman’s rank correlation matrix between clinical outcomes and gait/balance parameters in the experimental group. Each cell reports the correlation coefficient with significance indicated by asterisk). Red cells indicate positive correlations, blue cells negative correlations and white cells values close to zero. *p < 0.05, **p < 0.01, ***p < 0.001Legend: CT = Cadence; AGS = Average Gait Speed; GDI/R = Gait Deviation Index Right; GDI/L = Gait Deviation Index Left; GPS/R = Gait Profile Score Right; GPS/L = Gait Profile Score Left; SL/R = Step Length Right; SL/L = Step Length Left; COPL = Center of pressure- Path length; COPV = Center of pressure- Average velocity; COPL-CE = Center of pressure- Path Length- closed eyes; COPV- CE = Center of pressure- average velocity- closed eyes; Accuracy-CE = accuracy – closed eyes; AF/R = Average forces right; AF/L = Average forces left;AFR-CE = Average_forces_dx_eyes_closed; AFL-CE = Average_forces_sx_eyes_closed.
(PDF)

S1 File SPIRIT checklist. (TIFF)

S2 File Protocol. (PDF)

## Additional information

Correspondence and requests for materials should be addressed to Corresponding Author
