# Supplementary material for: Enhancing gait training with anti-gravity treadmill ‘Alter-G’ in patients with Parkinson’s disease
Source: PLoS One. 2026 Feb 25;21(2):e0341021. doi: 10.1371/journal.pone.0341021 (PMC12935195; doi:10.1371/journal.pone.0341021)
Supplement: S1 Fig — Each cell reports the correlation coefficient with significance indicated by asterisk). Red cells indicate positive correlations, blue cells negative correlations and white cells values close to zero. *p < 0.05, **p < 0.01, ***p < 0.001Legend: CT = Cadence; AGS = Average Gait Speed; GDI/R = Gait Deviation Index Right; GDI/L = Gait Deviation Index Left; GPS/R = Gait Profile Score Right; GPS/L = Gait Profile Score Left; SL/R = Step Length Right; SL/L = Step Length Left; COPL = Center of pressure- Path length; COPV = Center of pressure- Average velocity; COPL-CE = Center of pressure- Path Length- closed eyes; COPV- CE = Center of pressure- average velocity- closed eyes; Accuracy-CE = accuracy – closed eyes; AF/R = Average forces right; AF/L = Average forces left;AFR-CE = Average_forces_dx_eyes_closed; AFL-CE = Average_forces_sx_eyes_closed. (PDF) [file pone.0341021.s001.pdf]

Study Title

"Gait Training with the AntiGRAVIty TREadmill 'Alter-G' in Patients with Parkinson's Disease"

Protocol Code: GRAVITESON
Version No. 1 - Date: 03.12.2020
Participating Center: C.A.R.E.N. – Neurolesi Center "Bonino-Pulejo", Messina, Italy
Principal Investigators: Dr. Roberta Cellini, Dr. Giuseppe Di Lorenzo
Executive Investigators: [Roberta Cellini, Vincenzo Cimino, Santino Lo Vecchio, Maria Accorinti, Annalisa Lo Giudice, Jolanda De Caro, Antonio Leo]
Data Collection and Processing: [Roberta Cellini, Vincenzo Cimino, Santino Lo Vecchio, Maria Accorinti, Annalisa Lo Giudice, Jolanda De Caro, Antonio Leo, Giuseppe Paladina, Amelia Brigandì, Chiara Sorbera, Francesco Corallo, Giuseppa Maresca]
Operational Units: C.A.R.E.N., IRCCS Centro Neurolesi Bonino Pulejo, C.da Casazza, Messina
(Clinical Trial: NCT0574025) (https://clinicaltrials.gov/).

RATIONALE

Parkinson's Disease (PD) is a neurodegenerative disorder characterized primarily by motor symptoms—rigidity, bradykinesia, tremor—and postural instability, balance impairment, and gait deficits. As the disease progresses, cognitive and behavioral disturbances often emerge, significantly limiting both functional independence and social life.

Although pharmacologic treatments have modified the natural course of the disease, gait and balance disorders tend to worsen, leading to higher levels of disability. Postural instability results in falls, often due to sudden loss of balance and absent compensatory reflexes, leading to musculoskeletal injuries such as femoral fractures. Freezing of gait (FOG) is also a major contributor to falls.

Physiotherapy—including cueing strategies, treadmill training, and cognitive-motor techniques—has proven useful in improving gait and balance in PD. Among rehabilitation strategies, the **Multidisciplinary Intensive Rehabilitation Treatment (MIRT)**, which combines standard physical therapy with treadmill training enhanced by auditory and visual cues, has shown improvements in gait parameters (speed, cadence, stride length) and reduced FOG episodes. Early application of MIRT has also been associated with slowed disease progression and improved pharmacologic response.

In elderly patients (typically >65 years old), pain from comorbidities like osteoarthritis and osteoporosis complicates the rehabilitation process. Musculoskeletal pain is often underdiagnosed and undertreated, posing an obstacle to intensive rehabilitation.

A 2020 study by **Baizabal-Carvallo** found that low-intensity training programs improved FOG and mobility while reducing fall-related injuries.

The **Alter-G M320 antigravity treadmill**, originally developed by NASA, uses Differential Air

Pressure (DAP) to reduce body weight by up to 80%, enabling safe and biomechanically accurate

walking and running. Its uses include rehabilitation for elderly patients, post-surgical recovery, prosthetic adaptation, neurologic conditions (e.g., PD, MS), and weight loss without joint stress.

OBJECTIVES

Primary Endpoints:

- Assess improvements in gait cycle quality and FOG episodes
- Measure cadence, stride length, gait speed
- Evaluate fall risk reduction
- Assess prevention/treatment of musculoskeletal pain syndromes

Secondary Endpoints:

- Quality of life improvements
- Mood/anxiety/depression outcomes

STUDY DESIGN

This is a **non-interventional observational study** using standard clinical practices. Eligible subjects undergo baseline evaluations (T0): 3D gait analysis, postural exam, stabilometric analysis, neuropsychological tests. Participants will complete 20 rehabilitation sessions over 4 weeks (5 per week). The experimental group will use **Alter-G** instead of a standard treadmill. Post-treatment evaluations (T1) mirror baseline tests.

Data collected:

- MDS-UPDRS II and III
- Hoehn & Yahr staging
- Berg Balance Scale (BBS)
- Timed Up and Go Test (TUG)
- Tinetti, 6MWT, FES-I, 10MWT
- Pain VAS
- Neuropsychological assessments (EQ-5D, PDQ-39, HAM-A, HADS)

Postural and 3D Gait Analysis:
Postural assessment via **Souchard Method (RPG®)**, including:

- Static/dynamic baropodometric exam
- Stabilometric test
- BTS Gait analysis system

STUDY DURATION

12 months. The sample includes 40 patients (20 experimental + 20 control). Controls will receive MIRT without Alter-G.

POPULATION

Sample Size:
20 patients with PD, aged 50–85, Hoehn & Yahr stages II–III, MMSE ≥ 24, with FOG but without musculoskeletal or cardiopulmonary contraindications. 20 matched controls. A simple randomization procedure was used to assign the 40 participants in a 1:1 ratio to the experimental group (n = 20) or the control group (n = 20), such that each participant had an equal and independent probability of allocation to either group

Inclusion Criteria:

- Clinical diagnosis of PD per MDS criteria
- Evidence of FOG
- Age 50–85
- H&Y stage II–III
- MMSE ≥ 24 (age- and education-adjusted)
- Written informed consent

Exclusion Criteria:

- Atypical parkinsonism
- MMSE ≤ 24
- Cardiac devices or infusion pumps
- Significant comorbidities contraindicating physical training
- Uncorrected vision/hearing loss
- No informed consent

STATISTICAL ANALYSIS

Descriptive and inferential statistics using **R software**.

- Shapiro-Wilk test for distribution
- Paired t-test or Wilcoxon for intra-group
- Independent t-test or Mann-Whitney for inter-group
- Pearson/Spearman correlations
- Significance: $p < 0.05$

APPROVAL ETHIC COMMITTEE

Transmission of the Ethical Committee Opinion from the IRCCS Sicilia Neurolesi "Bonino-Pulejo" Center for the clinical study titled: "Gait Training with the antiGRAVlty TREadmiII 'AIter-G' in patients with Parkinson's Disease." Study Code: GRAVITESON

Approved Opinion Register: 46/2020

ETHICS

- Conducted under GCP, local regulations, and the **Declaration of Helsinki**
- Informed consent process clearly outlined
- Data protection compliant with **GDPR (EU 2016/679)**
- Subjects anonymized with unique codes
- Every information about the protocol can request to corresponding author

ADMINISTRATIVE & LEGAL

- Awaiting Ethical Committee approval
- No external funding or compensation
- Covered by appropriate insurance
- Results to be publicly disseminated

FUNDING

This study was supported by Current Research Funds 2025, RRC-2025-23686388 Ministry of Health, Italy. The funding funding was assigned to IRCCS Centro Neurolesi Bonino Pulejo, Messina
