# Supplementary figures and images for: Enhancing gait training with anti-gravity treadmill ‘Alter-G’ in patients with Parkinson’s disease
Source: PLoS One. 2026 Feb 25;21(2):e0341021. doi: 10.1371/journal.pone.0341021 (PMC12935195; doi:10.1371/journal.pone.0341021)

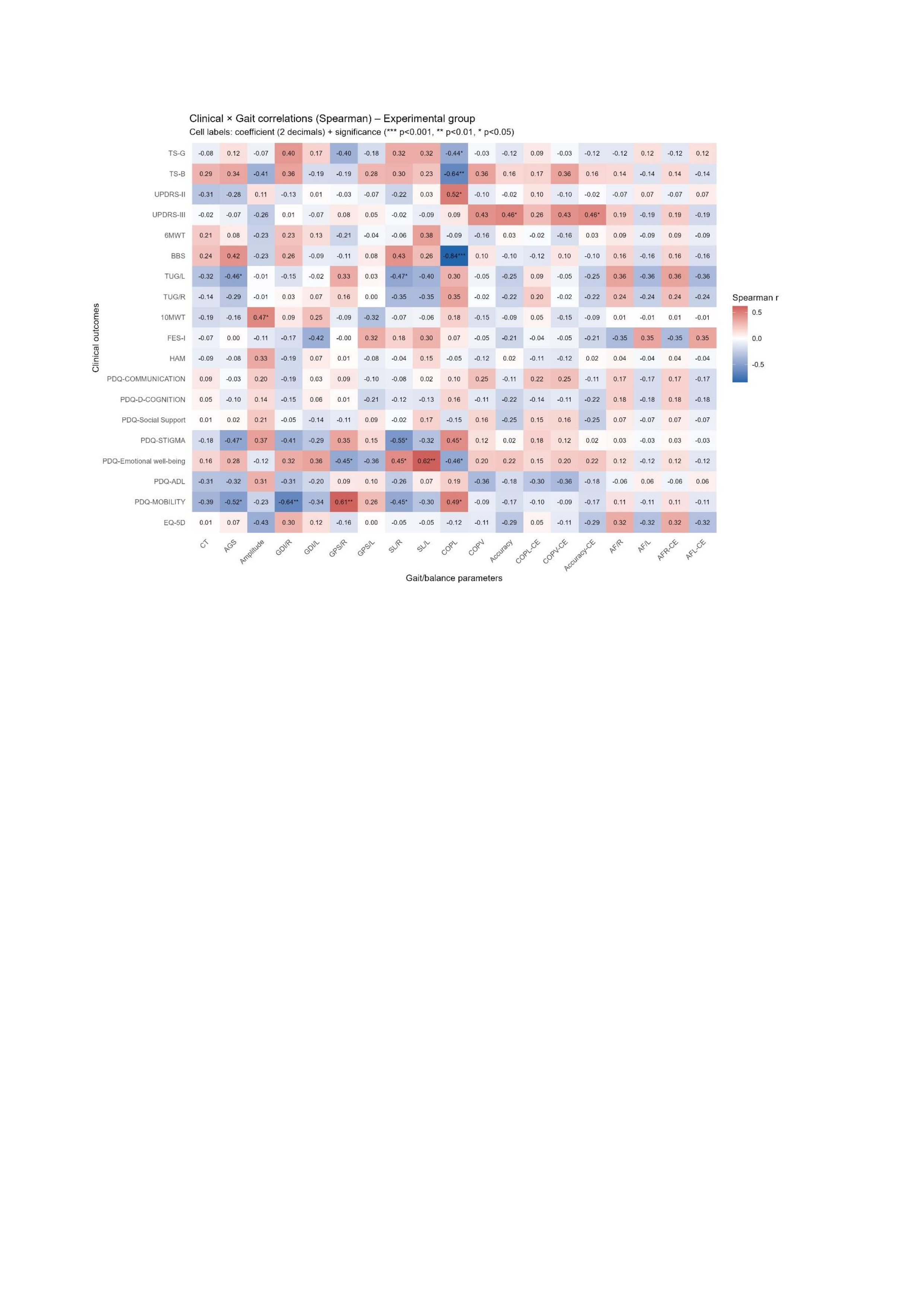

Supplement: S1 File — (TIFF) [file pone.0341021.s002.tiff]
